# Efficient identification of multiple pathways: RNA-Seq analysis of livers from ^56^Fe ion irradiated mice

**DOI:** 10.1186/s12859-020-3446-5

**Published:** 2020-03-20

**Authors:** Anna M. Nia, Tianlong Chen, Brooke L. Barnette, Kamil Khanipov, Robert L. Ullrich, Suresh K. Bhavnani, Mark R. Emmett

**Affiliations:** 1 Biochemistry and Molecular Biology, The University of Texas Medical Branch, Galveston, Texas USA; 2 Institute for Translational Sciences, The University of Texas Medical Branch, Galveston, Texas USA; 3 Pharmacology and Toxicology, The University of Texas Medical Branch, Galveston, Texas USA; 4 Radiation Effects Research Foundation, Hiroshima, Japan; 5 Radiation Oncology, The University of Texas Medical Branch, Galveston, Texas USA

**Keywords:** WGCNA, Modularity, Gene expression profiling, RNA-seq, Sequence analysis, Modularity maximization, Network visualization

## Abstract

**Background:**

mRNA interaction with other mRNAs and other signaling molecules determine different biological pathways and functions. Gene co-expression network analysis methods have been widely used to identify correlation patterns between genes in various biological contexts (e.g., cancer, mouse genetics, yeast genetics). A challenge remains to identify an optimal partition of the networks where the individual modules (clusters) are neither too small to make any general inferences, nor too large to be biologically interpretable. Clustering thresholds for identification of modules are not systematically determined and depend on user-settable parameters requiring optimization. The absence of systematic threshold determination may result in suboptimal module identification and a large number of unassigned features.

**Results:**

In this study, we propose a new pipeline to perform gene co-expression network analysis. The proposed pipeline employs WGCNA, a software widely used to perform different aspects of gene co-expression network analysis, and Modularity Maximization algorithm, to analyze novel RNA-Seq data to understand the effects of low-dose ^56^Fe ion irradiation on the formation of hepatocellular carcinoma in mice. The network results, along with experimental validation, show that using WGCNA combined with Modularity Maximization, provides a more biologically interpretable network in our dataset, than that obtainable using WGCNA alone. The proposed pipeline showed better performance than the existing clustering algorithm in WGCNA, and identified a module that was biologically validated by a mitochondrial complex I assay.

**Conclusions:**

We present a pipeline that can reduce the problem of parameter selection that occurs with the existing algorithm in WGCNA, for applicable RNA-Seq datasets. This may assist in the future discovery of novel mRNA interactions, and elucidation of their potential downstream molecular effects.

## Background

RNA-Seq, an approach to genome profiling that uses deep-sequencing technologies, has become an increasingly common technique to understand biological phenomena at the molecular level. This method generates quantitative count data on thousands of different mRNAs within each experiment. Comparing the expression of genes between different experimental conditions identifies hundreds of differentially expressed genes, but translating these lists into key functional distinctions between conditions has proved challenging. Since there are thousands of genes in each sample, many researchers filter their gene lists based on different criteria, in order to extract meaningful biological information. One such filtering criteria is based on differential gene expression analysis. Differential gene expression analysis has traditionally been used to determine genes that are statistically significantly differentially expressed between different experimental conditions based on different metrics, such as non-parametric generalized linear models, independent sample t-tests, and log_2_ fold changes [1]. Even though differential gene expression analysis is one of the most common methods for identifying disease pathways in various experimental conditions, it does not take into consideration the interactions of genes that work as a system to coordinate cellular functions. As a result, using only differential gene expression analysis would limit mechanistic interpretations of the data. mRNAs never act in isolation, but rather in concert with each other and other signaling molecules to define a particular biological pathway and function. Interactions of these signaling molecules can be viewed as networks of interconnected genes and their partners, that are up/down regulated under certain chemical or environmental conditions.

Many algorithms that utilize network theory have found applications in identifying and analyzing these molecular interactions [2–4]. Correlation networks are an example of such algorithms, and describe the co-expression of many genes in response to changing conditions, which can ultimately provide information about the underlying molecular mechanisms or biochemical pathways [5, 6]. In particular, the Weighted Gene Co-expression Network Analysis (WGCNA) method, which is provided as an R software package, has been widely used for performing different aspects of weighted correlation network analysis [7]. The co-expression networks used in WGCNA are constructed based on correlations between the quantitative measurements of each gene, and can be described by an *n x m* matrix *X = [x*_*ij*_*]*. Here the row indices (i = 1,…,n) correspond to different genes, and the column indices (j = 1,…,m) correspond to different sample measurements. While co-expression networks integrate systems-level information to provide a mechanistic interpretation of the dataset, detecting modules (clusters) of closely related mRNAs within the co-expression networks has been a challenging problem. Significant pathways that are identified by different clustering methods often yield tens or hundreds of genes, making biological interpretation and validation challenging. Further, many clustering techniques such as Dynamic Tree Cut utilized in WGCNA rely on user-settable parameters, including minimum module size, and are sensitive to cluster splitting [8, 9]. While many of these module detection methods perform optimally on some datasets, they may fail to effectively detect patterns in other datasets. A practical challenge in terms of discovering modules and determining the total number of modules is the identification of the optimal number of modules in the network, such that the individual modules are neither too large, preventing meaningful interpretation, nor too small, allowing little to no general inference. In general, characterizing and detecting community structures within networks has been a challenging problem in the study of networks [10–12]. One of the most commonly used metrics to investigate community structure is a quality index for clustering known as Modularity [13–15]. In spite of its popularity, Modularity does have drawbacks. The resolution limit (RL) problem is one of the most significant drawbacks, referring to the problem of maximizing Modularity while hindering one’s ability to detect communities that contain fewer links [16]. To address this problem, several approaches have been introduced [17–20]. Of these approaches, Modularity Maximization, which utilizes modularity density measures, has been shown to eliminate rather than merely reduce the RL problem in a wide range of networks [20].

In this study, we propose a pipeline using Modularity Maximization [20] to effectively detect and evaluate modules from co-expression networks obtained from the adjacency matrix, utilizing WGCNA [4, 7]. We employ the above technique to characterize the effects of ^56^Fe irradiation on mice livers, in order to study the potential consequences of deep space travel. In particular, astronauts will be exposed to high-charge, high-energy ions (HZE) during deep space travel. Even at low doses, exposure to HZE can lead to cancer [21, 22]. However, the effects of ions found in the deep space environment on cancer formation is not well understood since very few people have been exposed to space irradiation. As human exploration into deep space increases in the future, characterization of and intervention in irradiation-induced diseases will become more important. Previous studies have shown that irradiation of mice with low-dose HZE, specifically ^56^Fe ions, significantly increases the incidences of hepatocellular carcinoma (HCC) [23, 24]. HCC is the most common type of liver cancer, and its formation has mainly been studied in the context of terrestrial risk factors such as chronic hepatitis B/C virus infection, exposure to aflatoxin, obesity, smoking, and heavy alcohol consumption [25–27]. However, there is limited knowledge of the effects of low-dose ^56^Fe ion irradiation on the formation of HCC. To better understand the molecular mechanisms of low-dose ^56^Fe induced HCC, we used RNA-Seq to determine gene expression changes in the hepatic micro-environment of ^56^Fe ion irradiated compared to non-irradiated control mice at 5 different time points post-irradiation. We hypothesized that mitochondrial pathways could be significantly affected, since mitochondria represent a substantial cellular target volume (4–25% depending on the cell) [28]. In this manuscript, we will describe how WGCNA can be integrated with Modularity Maximization to construct co-expression correlation networks of differentially expressed genes and detect modules using data obtained from RNA-Seq.

## Results

### Differential gene expression analysis

Results of differential gene expression analysis are shown in Table 1, which includes the total number of differentially expressed genes at each time point, as well as whether genes are up/down-regulated.

**Table 1:** Results of differential gene expression analysis of RNA-Seq data from ^56^Fe Irradiated and non-Irradiated control mice livers at various time points analyzed using edgeR package.

Differentially Expressed Genes				
Comparison	Time	Total # of Differentially Expressed Genes	Up Regulated	Down Regulated
^56^Fe Irradiated/Non-Irradiated Control	1 month	645	322	323
^56^Fe Irradiated/Non-Irradiated Control	2 months	914	637	277
^56^Fe Irradiated/Non-Irradiated Control	4 months	497	259	238
^56^Fe Irradiated/Non-Irradiated Control	9 months	704	498	206
^56^Fe Irradiated/Non-Irradiated Control	12 months	285	75	210
^56^Fe Irradiated/Non-Irradiated Control	Sum	3045	1791	1254

### Feature selection

A total of 2273 unique differentially expressed genes were identified in comparison between ^56^Fe irradiated and non-irradiated controls. Genes that were statistically significant with FDR ≤ 10^− 5^ were used for downstream network analysis; 487 unique genes met the filtration criteria. The significance cut off can be adjusted to a higher value if a researcher decides to investigate more genes, depending on the study goals, experimental conditions, and data variability.

### WGCNA

We initially used the WGCNA Dynamic Tree Cut algorithm [7] to identify modules within the selected differentially expressed genes. Module identification with this algorithm requires two parameters to be determined prior to network construction: deepSplit, and minClusterSize. deepSplit can be either logical or an integer in the range 0 to 4. It controls the sensitivity to cluster splitting. Higher values result in smaller clusters. minClusterSize represents the minimum number of genes needed in a module to be considered a separate module. Table 2 shows the results of WGCNA module identification using different minClusterSize values, with a default deepSplit value of 2. As minClusterSize increases, the total number of modules decreases. These values produce different types of networks with differing numbers of unassigned genes. If a gene does not belong to a specific module, it is assigned to the Grey/Unassigned Module. The number of unassigned genes varied between 36 and 73 (shown in the last column of Table 2). At the same time, the total number of modules needs to be within a reasonable range, in order to be able to meaningfully investigate the relationship between genes; 70 different modules each containing a few genes may not provide meaningful information about these co-expression patterns. In our dataset, networks with a total of 11–18 modules provided interpretable co-expression patterns for further investigation, using pathway analysis tools as well as experimental validation. However, these clustering parameters resulted in 61–69 unassigned genes, representing ~ 12–14% of the 487 selected highly significant features.

**Table 2:** WGCNA Results with Dynamic Tree Cut Algorithm: deepSplit provides a rough control over the sensitivity to cluster splitting. The higher the value (or if TRUE), the more and smaller clusters will be produced. The Dynamic Tree Cut may identify modules whose expression profiles are very similar. The parameter minClusterSize allows one to control the minimum number of genes in a module, helping to avoid having similar clusters of few genes. As shown in the table, the lower values of minClusterSize increase the ‘Total Number of Modules’. Moreover, as this number increases, the ‘Number of Genes in Unassigned Module (Grey)’ increases as well

WGCNA Results			
minClusterSize	deepSplit	Total Number of Modules	Number of Genes in Unassigned Module (Grey)
2	2	70	36
3	2	49	37
4	2	37	46
5	2	31	49
6	2	25	57
7	2	20	60
8	2	18	61
9	2	17	65
10	2	15	67
11	2	15	67
12	2	11	69
13	2	11	69
14	2	9	73

### WGCNA with Modularity Maximization

To optimize the number and size of identified modules as well as reduce the number of unassigned genes (~ 12–14%), we exploited the concept of Modularity Maximization, to assist in finding community structures, as an alternative to utilizing the Dynamic Tree Cut algorithm employed in the standard WGCNA pipeline. Dynamic Tree Cut relies on hierarchical clustering, which is based on the relative distance between genes and samples. Modules are detected by “cutting” these trees, which can lead to many different small modules or a few large modules, depending on the selection of the minClusterSize and deepSplit parameters. Using Modularity Maximization, we were able to identify modules without the need to set these parameters empirically. In particular, the adjacency matrix with a soft threshold beta of 16 (corresponding to R^2^ = 0.9) was first computed using WGCNA, then a clustering algorithm based on Modularity Maximization was used to automatically find community structures in our dataset. We chose the Modularity Maximization method, since the metric of Modularity has been widely used to detect and assess community structures in social and biological networks since its inception [20, 29–32].

**Fig. 1:**
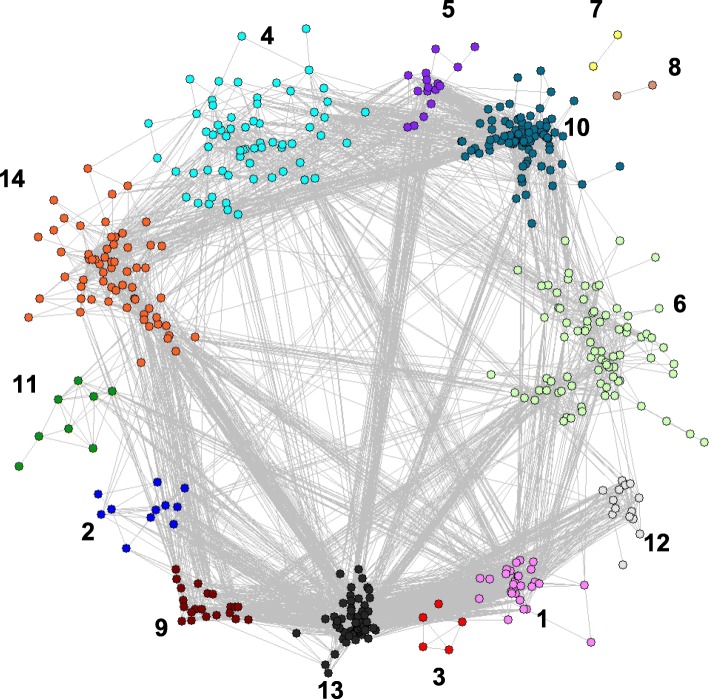
Modularity Maximization Network. Modules identified by performing Modularity Maximization on the network obtained from WGCNA. The module numbers on the network correspond to the modules shown in Table 3. A total of 14 genes were unassigned

Utilizing the modularity-based clustering algorithm to identify modules, 14 modules were discovered, and only 14 individual genes were unassigned. The final modularity score was Q = 0.696, which is indicative of a strong modular structure in the network. Figure 1 depicts the 14 modules in the network, and Table 3 shows the number of genes included in each module along with the enriched molecular pathways, as discussed below.

**Table 3:** Ingenuity Pathway Analysis on individual modules

Pathway Analysis		
Module #	Genes #	Molecular Pathways Identified as Enriched (*p-*value ≤ 0.05) in Each Module
**1**	**28**	Sirtuin Signaling Pathway, Mitochondrial Dysfunction, Oxidative Phosphorylation, LXR/RXR Activation, FXR/RXR Activation, NAD Biosynthesis III, Oleate Biosynthesis II, Histamine Degradation
**2**	**11**	IL-9 Signaling, Transcriptional Network in Embryonic Stem Cells, Mitotic Roles of Polo-Like Kinase, GM-CSF Signaling, Growth Hormone Signaling, JAK/STAT Signaling, STAT3 Pathway
**3**	**5**	No Pathway. 3 genes in this module are not Identified. Specifically, Gm28437, Gm28661, Gm29216. The other two are mir-122 (microRNA 122) and Gm10925 (ATP Synthase F0 subunit 6)
**4**	**65**	Acyl-CoA Hydrolysis, Stearate Biosynthesis I, Pregnenolone Biosynthesis, Histidine Degradation VI, Ubiquinol-10-Biosynthesis, Asparagine Biosynthesis I, a-tocopherol Degradation, LSP/IL-1 Mediated Inhibition of RXR Function, FXR/RXR Activation
**5**	**16**	Toll-like Receptor Signaling, Heme Degradation, IL-12 Signaling and Production in Macrophages, Acute Phase Response Signaling, Granulocyte Adhesion and Diapedesis, NF-kB Signaling, Agranulocyte Adhesion and Diapedesis, Production of Nitric oxide and ROS in Macrophages
**6**	**80**	Nicotine Degradation II, Glutathione-mediated Detoxification, Circadian Rhythm Signaling, LPS/IL-1 Mediated Inhibition of RXR Function, Nicotine Degradation III, Adipogenesis Pathway, PXR/RXR Activation, Melatonin Degradation I
**7**	**2**	No Pathway. Two genes (CYP26A1 and CYP26B1) are both part of cytochrome P450 family 26 subfamily A member 1 and subfamily B member 1. They are involved in Pregnenolone Biosynthesis, Histidine Degradation VI, Ubiquinol-10 Biosynthesis and RAR Activation
**8**	**2**	No Pathway. Two genes (ANGPTL8 and HES1). HES1 is involved in Notch Signaling, VDR/RXR Activation.
**9**	**21**	Unfolded protein response, Protein Ubiquitination Pathway, eNOS Signaling, Glucocorticoid Receptor Signaling, Endoplasmic Reticulum Stress Pathway (6 genes are heat shock proteins)
**10**	**89**	Acute Phase Response Signaling, IL-10 Signaling, IL-6 Signaling, Role of Macrophage, Fibroblasts and Endothelial Cells in Rheumatoid Arthritis, LXR/RXR Activation, B Cell Receptor Signaling, Altered T Cell and B Cell Signaling in Rheumatoid Arthritis, Hepatic Cholestatis
**11**	**8**	No Pathway. 4 unidentified genes (Cm23935, Gm24187, Rn 18 s-rs5, Gm155644) and other 4 (Leucyl-tRNA synthetase 2, microRNA 6236, s-rRNA, l-rRNA)
**12**	**14**	No Pathway, basic helix-loop-helix family involved in Circadian Rhythm Signaling, Mir17hg, Small nuclear RNA (Snora57, Snora78) and 10 unidentified genes.
**13**	**69**	Estrogen-mediated S-phase Entry, Cell Cycle Regulation, Chronic Myeloid Leukemia Signaling, a-tocopherol Degradation
**14**	**63**	NRF2-mediated Oxidative Stress Pathway, Endoplasmic Reticulum Stress Pathway, Unfolded Protein Response, Death Receptor Signaling, RhoA Signaling, FXR/RXR Activation.

### Module validation and properties

**Fig. 2:**
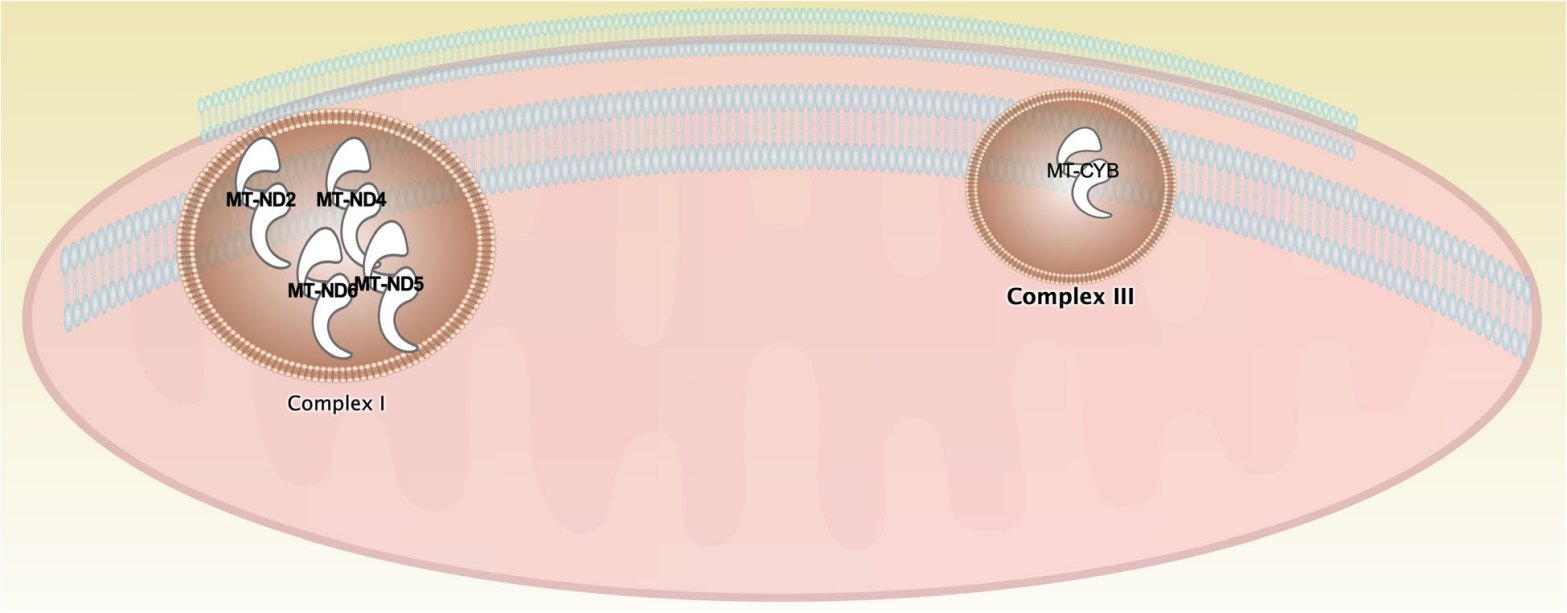
Mitochondrial Dysfunction Pathway Genes. Five of the genes from module 1 are involved in the mitochondrial dysfunction pathway. Specifically, 4 of them, MT-ND2, MT-ND4, MT-ND5, and MT-ND6 are different subunits of Complex I. MT-CYB, or cytochrome b is part of Complex III/bc which also regulates Complex I. Figure was made using Ingenuity Pathway Analysis (IPA), (QIAGEN Inc., Hilden, Germany)

**Fig. 3:**
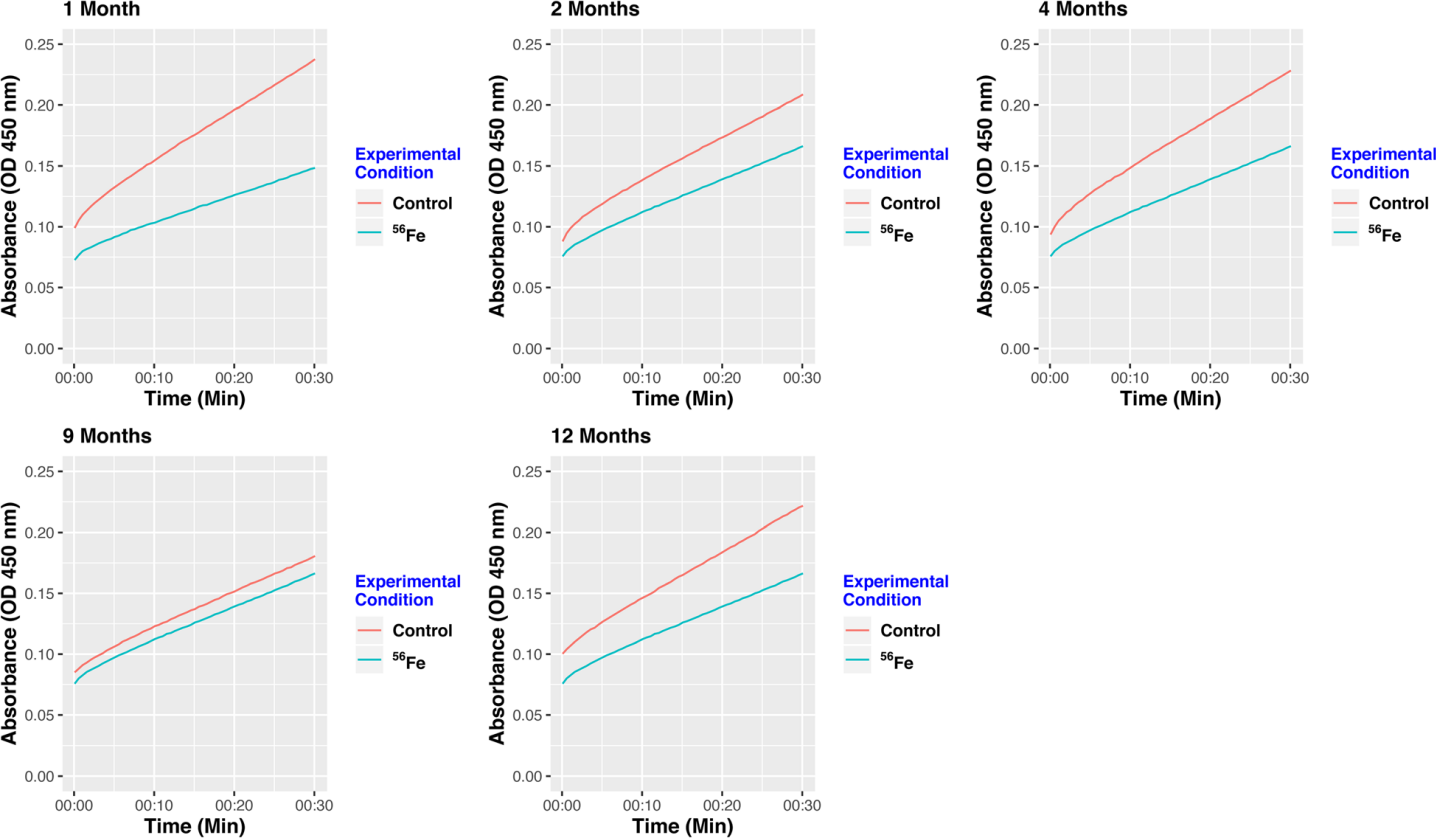
Results of Mitochondrial Complex I Functional Assay performed for each time point. Complex 1 activity was decreased after exposure to ^56^Fe irradiation as compared to non-irradiated control at each time point

To explore the biological relevance of the modules, each module was investigated by Ingenuity Pathway Analysis (IPA). Specifically, module 1 was shown to be significant (−log_10_(*p-value*) ≥ 1.3) in mitochondrial pathways, such as the Sirtuin Signaling Pathway, Mitochondrial Dysfunction, and Oxidative Phosphorylation [31]. All the genes involved in mitochondrial dysfunction in our dataset were contained in module 1. In particular, Fig. 2 shows that 5 of these genes express different subunits of mitochondrial complex I and III. To validate these results, we performed an additional experimental technique to determine whether complex I activity is reduced in response to ^56^Fe irradiation. Complex I activity was observed to be decreased in response to exposure to ^56^Fe HZE ion across all time points as measured by mitochondrial complex I enzyme activity (Fig. 3). The downstream effects of irradiation on mitochondrial functions have been emphasized [33], as mitochondria have been shown to occupy a substantial fraction of the cell volume [28]. Therefore, they may be fairly easily targeted by irradiation as the ^56^Fe nuclei traverse the cell. The electron transport chain in the mitochondrion is composed of five protein complexes (I-V) that perform a series of oxidation-reduction reactions, in which O_2_ is the final electron acceptor and is reduced to a water molecule. One of the consequences of this process is the formation of reactive oxygen species (ROS), which is thought to arise from the leakage of electrons, specifically from complex I and III, and to a minor extent complex II [34–36]. Using oxygen as the final electron acceptor causes mitochondria to consume about 90% of the body’s oxygen but also become the richest source of ROS [36–39]. The upregulation of mitochondrial genes shown in Fig. 2, specifically in complex I and complex III, suggests that leakage of electrons from these two complexes results in increased Complex I and III enzyme activity. This leads to further the overexpression of these genes in response to ^56^Fe irradiation. Other modules could potentially be validated in future experimental designs, involving live animals and more fresh tissues. For example, module 2 can be tested for JAK/STAT signaling. STATs are ubiquitously expressed and mainly activated after stimulation of cytokine receptors. STATs function in the nucleus, but they are first activated in the cytoplasm and have then to be transported into the nuclear compartment [40]. This translocation can be assessed by indirect immunofluorescence. Additionally, STAT signaling can be experimentally validated by pharmacologically inhibiting STAT pathways with specific STAT inhibitors. Similarly, module 9 could be tested for Endoplasmic Reticulum (ER) stress pathways. Several molecular indicators of ER stress could be examined by Western Blots and/or proteomic analysis, which could demonstrate increased or decreased phosphorylation of ER stress proteins.

## Discussion

One of the current statistical challenges in identifying co-expression patterns in RNA-Seq data is a robust determination of the number and size of modules appropriate, across a variety of datasets. The choice of an appropriate clustering algorithm that yields the most biologically interpretable networks has been studied using different datasets and methods. For example, a study has investigated Recursive Indirect-Paths Modularity (RIP-M) for module detection in an RNA-Seq co-expression network. Using an influenza vaccine response study, the authors showed that RIP-M had higher cluster assignment accuracy as compared to Newman Modularity, and similar results to WGCNA [41]. We compared WGCNA, RIP-M, and the combined WGCNA-M method based on the Rand Index (RI) [29]. In calculating the RIs, we considered every unassigned gene, as a cluster by itself, since such genes are viewed as not being similar to each other. The RIs for WGCNA-M versus WGCNA, WGCNA-M versus RIP-M, and WGCNA versus RIP-M were 0.909, 0.892, 0.936, respectively. The numbers of genes unassigned to a cluster for WGCNA-M, WGCNA, and RIP-M were 14, 108, and 0, respectively. All of the 14 genes which were unassigned in WGCNA-M were also unassigned in WGCNA. Based on our observed RIs, RIP-M and WGCNA were the most similar when applied to our dataset. Like WGCNA, RIP-M also requires the parameter minModuleSize (minClusterSize) as well as an additional parameter, maxModuleSize, which specifies a target range of module sizes. All genes assigned to a module below minModuleSize are then grouped together and merged into a further module. Modules above maxModuleSize are split in subsequent iterations to arrive at the target range. RIP-M forces all genes to be assigned to a cluster; the 14 genes unassigned by WGCNA and the WGCNA-M approach for our dataset were placed into cluster 1 by RIP-M. Community detection method selection is an important issue in cluster analysis and may greatly influence the results of a study and their biological interpretability. Therefore, it is imperative to select the most suitable method for each specific experimental design, and for the nature of the data being investigated. A complete list of gene cluster-assignments for each method is provided in Supplemental Table 1.

Utilizing Modularity Maximization to detect community structures provides an additional way to construct a network and explore various RNA-Seq datasets; however, WGCNA-M is not limited to this application domain, and can be applied to detect co-expression patterns amongst other omics studies. Protein or lipids can be linked together in networks via a defined functional relationship in a similar fashion. Methodologically, MS-based proteomics and lipidomics tend to have consistency, and coverage issues [42–47] as compared to RNA-based high throughput methods. As a result, some network analysis methods as applied to proteomic data may not capture the complexity and nuances underlying biological processes, and alternative approaches may be needed to complement the existing analytical tools. Similar to any other analytical method, the network-based WGCNA-M analysis method must be applied appropriately based on the inherent quality and nature of each dataset. This will allow us to gain robust biological insight and decipher the unique patterns in our data from which we can further understand the complexity and coordinated function of the system being investigated. In the current study, utilization of WGCNA with Modularity Maximization resulted in the identification of biologically interpretable and relevant modules, without the need for parameter optimization.

## Conclusions

In this study, we proposed a new pipeline that combines the adjacency matrix notion of WGCNA with Modularity Maximization to find modules that are involved in specific biological pathways. To show the validity of the identified modules, we conducted gene enrichment analysis and experimental validation. Our results showed that mitochondrial pathways that were changed in response to irradiation were contained in the same module. Further, our data indicates that even after performing stringent feature selection focusing on significant genes (FDR ≤ 10^− 5^), WGCNA-M was still able to identify biologically relevant modules. The use of the WGCNA Dynamic Tree Cut clustering algorithm in our dataset resulted in a high number of unassigned genes (61–69). On the other hand, WGCNA-M reduced the number of unassigned genes to 14 while maintaining an optimal number of modules/specific pathways. The proposed pipeline enables the identification of network and community structures without requiring optimization of the minClusterSize and deepSplit parameters. The increasing number of high throughput genomic datasets, together with the use of appropriate network pipelines, will enable researchers to efficiently investigate molecular mechanisms and pathways involved in different disease processes.

## Methods

In this section, we describe the WGCNA combined with Modularity Maximization for community detection pipeline used in the RNA-Seq dataset. Our evaluation strategy was targeted to analyze data from an RNA-Seq experiment of ^56^Fe irradiated and non-irradiated control mice liver lobes, designed to characterize the microenvironmental changes induced by HZE irradiation (similar to HZE ions encountered in deep space travel,) and that lead to induction of HCC. Our aim is to detect modules (clusters of genes) that are related by correlation across samples, and differ between experimental conditions. The resulting co-expression networks were analyzed using functional enrichment analysis and experimentally validated.

### Animal experiments and sample preparation

C57BL/6NCrl mice purchased from Charles River (Wilmington, MA) were used in this experiment. Tumor induction studies and studies of molecular changes in the irradiated tissues can only be conducted in whole animals. Further, based on an extensive literature search and examination of studies previously approved by the institutional animal care and use committees (IACUCs), computer models or cell culture studies are not possible. The numbers of animals used were based on the expected numbers of irradiation-related tumors that would develop if animals were allowed to live out their lifespans. Power calculations for numbers in this study are based on the chi-square test for comparing two proportions, with a two-sided significance level set at 0.05 at 80% power.

The serial sacrifice study included 15 male mice with 3 mice per time point at five time-points (30, 60, 120, 270, and 360 days) post-exposure to HZE, specifically ^56^Fe irradiation. Additionally, 15 mice were used as controls, at the same time points, resulting in a total of 30 mice for this study. The two groups were: 600 Me V/n ^56^Fe (0.2 Gy) and non-irradiated/sham-irradiated control. The mice were shipped from the vendor to Brookhaven National Laboratories (BNL) and housed at the BNL animal facility until the time of irradiation at the NASA Space Radiation Laboratory (NSRL). Following irradiation, the animals were shipped to the UTMB Animal Resources Center (ARC), quarantined for 1 month, and maintained in the ARC for the duration of the experiment. Animals were housed in sterile cages with free access to food and water. Facilities at both BNL and UTMB are fully AAALAC accredited, ensuring adequacy of animal care at both animal facilities.

At each of the five time-points, 3 animals from each group were randomly selected and euthanized using CO_2_ asphyxiation, as per current AVMA guidelines for euthanasia. Prior to euthanasia, animals were weighed and weights recorded. Post euthanasia, tissues of the left lobe of livers were collected, snap-frozen on either dry ice or liquid nitrogen, and stored at − 80 °C until tissues could be extracted for RNA analysis. Livers were sampled by taking two 40-μm thick slices using a cryotome at − 20 °C.

### Acquisition of RNA-Seq data

Total RNA was isolated from the liver slices using RNAqueous™ Total RNA Isolation Kit (ThermoFisher Scientific, Waltham, MA), and rRNA was removed using the Ribo-Zero™ rRNA Removal Kit (Illumina, San Diego, CA), prior to library preparation with the Illumina TruSeq RNA Library kit. Samples were sequenced in a paired-end 50 base format on an Illumina HiSeq 1500. FastQC was utilized for the quality evaluation of FASTQ files [48]. All FastQC reports were examined prior to the analysis of RNA-Seq samples. The total number of reads used in analysis varied between 23 and 35 million. A complete list of samples, and related reads information is available in Table 4.

**Table 4:** Sample List and Total Reads

Sample Information					
Number	Sample	Treatment Type	Time	Biological Replicate	Total Sequences
1.	H2	Non-Irradiated Control	1 month	1	32,905,344
2.	H3	Non-Irradiated Control	1 month	2	28,318,081
3.	H4	Non-Irradiated Control	1 month	3	27,220,319
4.	H7	Non-Irradiated Control	2 months	1	31,264,466
5.	H8	Non-Irradiated Control	2 months	2	31,375,164
6.	H9	Non-Irradiated Control	2 months	3	34,782,071
7.	H11	Non-Irradiated Control	4 months	1	24,449,063
8.	H12	Non-Irradiated Control	4 months	2	27,944,559
9.	H13	Non-Irradiated Control	4 months	3	23,137,137
10.	H16	Non-Irradiated Control	9 months	1	34,216,914
11.	H17	Non-Irradiated Control	9 months	2	30,149,494
12.	H18	Non-Irradiated Control	9 months	3	29,855,702
13.	H21	Non-Irradiated Control	12 months	1	26,910,777
14.	H22	Non-Irradiated Control	12 months	2	31,877,754
15.	H23	Non-Irradiated Control	12 months	3	33,432,277
16.	K2	^56^Fe Irradiated	1 month	1	31,868,688
17.	K3	^56^Fe Irradiated	1 month	2	37,890,611
18.	K4	^56^Fe Irradiated	1 month	3	25,953,453
19.	K6	^56^Fe Irradiated	2 months	1	47,994,834
20.	K7	^56^Fe Irradiated	2 months	2	34,603,257
21.	K8	^56^Fe Irradiated	2 months	3	32,128,695
22.	K12	^56^Fe Irradiated	4 months	1	27,386,313
23.	K13	^56^Fe Irradiated	4 months	2	29,914,981
24.	K14	^56^Fe Irradiated	4 months	3	28,626,258
25.	K16	^56^Fe Irradiated	9 months	1	24,669,187
26.	K17	^56^Fe Irradiated	9 months	2	24,014,552
27.	K18	^56^Fe Irradiated	9 months	3	28,179,114
28.	K23	^56^Fe Irradiated	12 months	1	28,350,658
29.	K24	^56^Fe Irradiated	12 months	2	31,439,904
30.	K25	^56^Fe Irradiated	12 months	3	25,132,399

Reads were aligned to the mouse GRCm38 reference genome using the STAR alignment program, version 2.5.3a, with the recommended ENCODE options [49]. The-quantMode GeneCounts option was used to obtain read counts per gene based on the Gencode release M14 annotation file [50].

### Differential gene expression analysis

Raw RNA-Seq data of 51,826 genes from 15 non-irradiated control and 15 ^56^Fe irradiated C57 mice liver tissue samples were subjected to differential gene expression analysis. All calculations and statistics were performed using statistical software R (R Foundation for Statistical Computing, Vienna, Austria) (version 3.5.1) [51]. Differential gene expression analysis was conducted using R software package edgeR [52, 53]. First, normalization factors were calculated to scale the raw library sizes. In addition, dispersion parameters based on generalized linear models (GLM) were estimated; in particular, the common dispersion for negative binomial GLMs, trended dispersion for negative binomial GLMs using the power method, and empirical Bayes tagwise dispersions for negative binomial GLMs [53, 54]. Statistical tests were then conducted for every time point, to compare between ^56^Fe irradiated and non-irradiated control samples, using a quasi-likelihood negative binomial generalized log-linear model for count data [55–57]. The Benjamini-Hochberg correction was applied, and genes with FDR ≤ 0.05 & fold change ≥1.5 (|(log_2_(Fold Change)| ≥ 0.59—up/down regulated) were extracted.

### Feature selection (FS)

Final differential gene expression analyses for all time points were combined. For genes differentially expressed at multiple time points, the lowest FDR was kept. The list was further filtered to keep only genes with FDR ≤ 10^− 5^. For the final selected gene list, raw variance stabilized normalized count data were retrieved from every RNA-Seq sample (*n* = 30) using the R package DESeq2 [58] This variance stabilized normalization method was specifically selected because it has proven useful for network construction using WGCNA methodology (The WGCNA FAQ).

### WGCNA

**Fig. 4:**
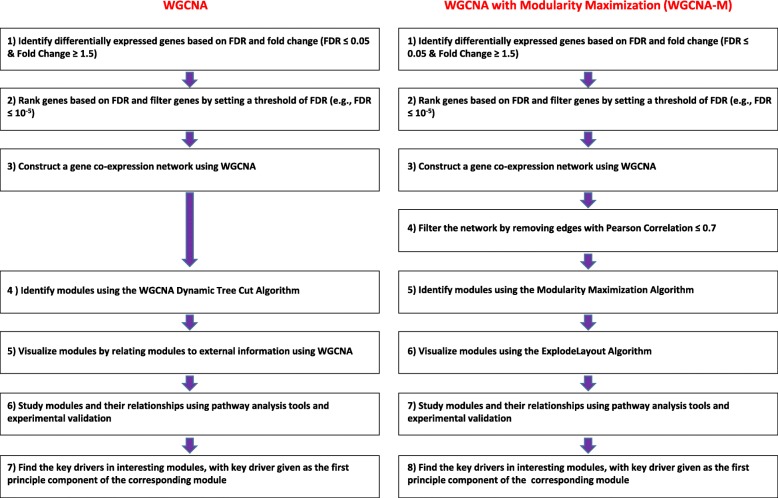
An overview of the WGCNA and WGCNA with Modularity Maximization (WGCNA-M) workflows

The gene expression profiles were comprised of 51,826 genes from 30 samples. Constructing a co-expression network on this original list without filtering could not meet a power threshold that corresponded to (R^2^ = 0.9) as recommended by WGCNA, and did not yield any biologically interpretable network. As a result, we first performed the feature selection based on differential gene expression analysis and FDR rank list (step 1–2 in Fig. 4, and described above) and then constructed the WGCNA network on genes given by this feature selection (step 3 in Fig. 4). WGCNA was performed on differentially expressed genes with FDR ≤ 10^− 5^ & fold change ≥1.5 (up/down-regulated). WGCNA analysis was performed per the methodology publication (step 4–7 in Fig. 4) [7].

### WGCNA with Modularity Maximization

**Fig. 5:**
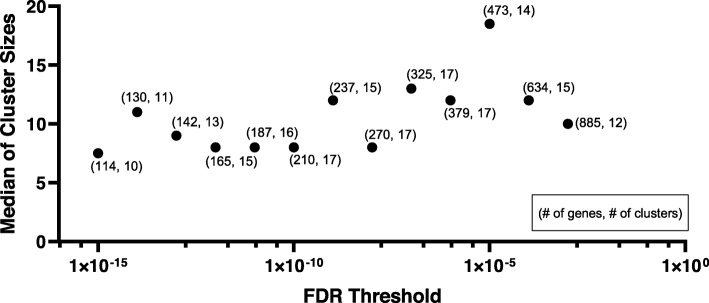
Plot to visualize different FDR thresholds using the Modularity Maximization Algorithm. The plot shows the change in the median number of clusters detected using Modularity Maximization, as the FDR cutoff is varied. The numbers next to each point designate the number of genes and the number of modules in the corresponding network. The module with the largest median size was chosen for further analysis, since small clusters are difficult to interpret

To evaluate the effect of feature selection on the median cluster size, we performed Modularity Maximization analysis on co-expression data derived by WGCNA applied to gene lists filtered over a range of FDR values. As shown in Fig. 5, the FDR value of 10^− 5^ led to the largest median cluster size, in this particular dataset. The features’ significance threshold can be optimized by plotting median cluster sizes at different FDR values. To derive clusters, the following steps were used. An adjacency matrix based on the Pearson correlation with the soft threshold was calculated by WGCNA [7]. The power threshold parameter was set to 16, corresponding to an R^2^ value of 0.9, which reflects a scale-free topology in which adjacency between all differential genes was calculated by a power function (step 3 in Fig. 4). The adjacency matrix was then filtered to only keep pairs of genes with a Pearson correlation of ≥0.7 (step 4 in Fig. 4). Then, module identification was performed using the Modularity Maximization clustering method (step 5 in Fig. 4) [20, 59]. Final modules were visualized using the ExplodeLayout algorithm (step 6 in Fig. 4) [60].

### Module statistical analysis

To determine whether the modules were observed by chance, the significance of the results was evaluated by comparing them to the average modularity of 1000 permutations of the weighted and thresholded co-expression network adjacency matrix. Each permutation of the network would preserve the number and weight of all the links but randomly shuffle them; thus it should still meet the scale-free network distribution criteria. Based on the 1000 permutations, we obtained a z-score of 86.8 for our modularity, indicating a strongly significant modular structure in the co-expression network as compared to random.

### Mitochondrial complex I enzyme activity assay

The mitochondria isolation kit for tissue (Abcam, ab110168) was used to isolate mitochondria from mice liver lobes. Complex 1 enzyme activity was monitored with a colorimetric microplate assay (Abcam, ab110168) using the isolated mitochondria from the liver.

### Functional Enrichment Analysis

To determine whether the co-expression modules were biologically meaningful, functional enrichment analysis was performed separately on every module. Significant functional pathways (−log_10_(*p-value*) ≥ 1.3) for each module were evaluated using Ingenuity Pathway Analysis (IPA) (QIAGEN Inc., Hilden, Germany) [31].

## Supplementary information

**Additional file 1: Supplementary Table 1.** Gene cluster-assignment comparison. Complete list of gene cluster-assignments using Recursive Indirect-Paths Modularity (RIP-M), Weighted Gene Co-Expression Network Analysis (WGCNA), and Weighted Gene Co-Expression Network Analysis with Modularity Maximization (WGCNA-M).

## Data Availability

The data discussed in this publication have been deposited in NCBI’s Gene Expression Omnibus (Nia et al., 2020) and are accessible through GEO Series accession number GSE136165 (https://www.ncbi.nlm.nih.gov/geo/query/acc.cgi?acc=GSE136165). The R and C scripts written for this publication are accessible through https://github.com/annamnia/Efficient-Identification-of-Multiple-Pathways-RNA-Seq-Analysis-of-Livers-from-56Fe-Ion-Irradiated-M

## Abbreviations

WGCNA: Weighted Gene Co-Expression Network Analysis
WGCNA-M: Weighted Gene Co-Expression Network Analysis with Modularity Maximization
HZE: High Charge High Energy Ions
IPA: Ingenuity Pathway Analysis
